# Timing and clinical risk factors for early acquisition of gut pathogen colonization with multidrug resistant organisms in the intensive care unit

**DOI:** 10.1186/s13099-024-00605-z

**Published:** 2024-02-21

**Authors:** Loren Shamalov, Madison Heath, Elissa Lynch, Daniel A. Green, Angela Gomez-Simmonds, Daniel E. Freedberg

**Affiliations:** 1 CUNY School of Medicine, 160 Convent Ave, New York, NY 10031 USA; 2 Department of Medicine, Columbia University Irving Medical Center-New York Presbyterian Hospital, New York, NY USA; 3 Division of Digestive and Liver Diseases, Columbia University Irving Medical Center-New York Presbyterian Hospital, New York, NY USA; 4 Clinical Microbiology, Department of Pathology and Cell Biology, Columbia University Irving Medical Center-New York Presbyterian Hospital, New York, NY USA; 5 Division of Infectious Diseases, Columbia University Irving Medical Center-New York Presbyterian Hospital, New York, NY USA

**Keywords:** Intensive care unit, Multidrug resistant Bacteria, Methicillin-Resistant Staphylococcus Aureus, Vancomycin-resistant Enterococcus, Extended-spectrum B-Lactamase resistance, Outcomes Research, Microbiome

## Abstract

**Background:**

Microbiome restitution therapies are being developed to prevent gut pathogen colonization among patients in the intensive care unit (ICU) and in other select populations. If preventive therapies are to be effective, they must be administered prior to pathogen acquisition. The timing and risk factors for early acquisition of gut pathogen colonization (within 72 h) are currently unknown and could be helpful to guide ICU trial design.

**Methods:**

This was a prospective cohort study. Patients in the ICU had deep rectal swabs performed within 4 h of ICU admission and exactly 72 h later. Early gut pathogen colonization was classified as the *new* presence (based on culture of rectal swabs) of one or more of the following organisms of interest: methicillin-resistant *Staphylococcus aureus* (MRSA), vancomycin-resistant (VRE), and Gram-negative bacteria that showed multidrug resistance (MDR) or third generation Cephalosporin resistance (Ceph-R). Clinical risk factors for early acquisition of gut pathogen colonization were captured using the Acute Physiology and Chronic Health Evaluation IV (APACHE IV) scoring system.

**Findings:**

Among 131 patients who were swabbed at ICU admission and 72 h later, the rates of gut pathogen colonization at ICU admission were 11.4%, 10.6%, 38.6%, and 8.3% for MRSA, VRE, MDR and Ceph-R Gram-negatives respectively. Among the patients who were negative for a given pathogen at ICU admission, the rates of early acquisition of gut pathogen colonization were 7.8% for MRSA (95% CI 3.6 to 14.2%), 7.7% for VRE (95% CI 3.6 to 14.1%), 11.3% for MDR Gram-negatives (95% CI 4.4 to 18.8%), and 4.2% for Ceph-R Gram-negatives (95% CI 1.4 to 9.5%). There were no clinical risk factors which independently predicted early acquisition of gut pathogen colonization.

**Interpretation:**

Early gut pathogen colonization was common in the ICU, but our single-center study could not identify any clinical risk factors which were significantly associated with acquisition of gut pathogens.

**Supplementary Information:**

The online version contains supplementary material available at 10.1186/s13099-024-00605-z.

## Introduction

Healthcare-associated infections caused by pathogenic (often MDR) bacteria are a significant concern in the medical ICU with mortality rates ranging from 26% to 80% [1]. Gut colonization with pathogenic bacteria is a risk factor for subsequent all-cause and pathogen-specific systemic infections [2–7]. Therefore, understanding the prevalence and dynamics of gut pathogen colonization is crucial for developing targeted interventions to mitigate the spread of these organisms and improve patient outcomes [8, 9]. 

Previous studies have demonstrated that the prevalence of gut pathogen colonization is high at the time of ICU admission and that many previously uncolonized patients acquire gut pathogens during prolonged ICU stays [3, 10–16]. Specific estimates of gut colonization and acquisition range based on institution and organism. For example, the prevalence of gut colonization with VRE at ICU admission has been estimated at 3.6–10.6%, with acquisition rates from 3.0 to 10.2% during the ICU stay [17, 18]. Similarly, for carbapenem-resistant Enterobacteriaceae (CRE), prevalence of gut colonization at the time of ICU admission has been estimated at 2.1–28% with acquisition rates reaching up to 58.6% during prolonged ICU stays [19, 20]. Many studies show strong associations between fecal carriage of MDR organisms and subsequent systemic infections with the same organisms: e.g., 77% of patients who developed MDR *Acinetobacter baumanii* infections were identified as fecal carriers [21, 22]. 

These prior studies are valuable but leave relatively wide estimates of confidence in rates of acquisition of gut pathogen colonization. Because many studies perform sampling at the end of the study period or immediately prior to ICU discharge, they do not always describe the dynamics of acquisition of gut pathogen colonization, which is crucial for the design of future ICU trials. Among ICU patients with sepsis, improvement or lack thereof during the early ICU period—typically defined as the first 72 h after ICU admission—has been shown to be a powerful predictor of subsequent risk for death [23, 24]. An underlying hypothesis of this study was that early gut pathogen colonization would similarly have greater importance for patient outcomes compared to late gut pathogen colonization.

There are additional gaps in the existing literature. Prior studies have determined the clinical risk factors for gut colonization with pathogenic bacteria, but they have not always distinguished between prevalent colonization (colonization at the time of ICU admission) and incident colonization (acquisition of gut pathogen colonization during some predetermined time period in the ICU) [25, 26]. Last, although prior studies of gut pathogen colonization in the ICU have often utilized protocolized sample collection—e.g., weekly after ICU admission—they have not always collected samples from the same individual patients, thus leaving some uncertainty related to whether there was a within-individual change in the gut pathogen colonization status of a given patient [19]. 

In this study, we performed protocolized collection of rectal swabs within 4 h of ICU admission (typically performed immediately when the patient was transferred into his or her ICU bed) and exactly 72 h later on the same patients. We used standard culture-based methods to define the within-individual changes in gut pathogen colonization status so that we could fully understand the acquisition rates and risk factors for gut pathogen colonization during the early period in ICU hospitalization. We focused on VRE, MRSA, and MDR Gram-negative (GN) bacteria because these organisms account for a large proportion of ICU-acquired infections and are readily cultured.

## Methods

### Population and rationale

The study enrolled adult patients aged 18 or more years who were newly admitted to an ICU (medical, surgical, or specialty ICU), provided informed consent for rectal swab collection (delayed patient or surrogate consent accepted) [27], and survived for a minimum of 72 h in the ICU after admission so that they could provide a follow-up rectal swab. Due to the time sensitive protocol, a “delayed consent” model was utilized, where baseline rectal swabs were obtained, and later sought permission to retain them [27]. The rationale for selecting 72 h as the sampling interval was based on prior ICU literature related to sepsis, including the APACHE studies [28, 29]. This literature suggests that ICU interventions must be deployed during the first 72 h to be most effective at altering patient outcomes. Because the focus of the study was on early acquisition of gut pathogen colonization, we excluded data from subjects who were swabbed at ICU admission and could not be swabbed again due to death or discharge. Our overall goal was to study a heterogeneous ICU population, the same population that would be targeted for future trials testing therapies to prevent acquisition of gut pathogen colonization.

### Rectal swabs

Rectal swabs were performed by an ICU nurse, within 4 h of ICU admission and then again 72 h later (+/- 4 h). The swab at admission was typically taken immediately as the patient transitioned from the transport gurney to the ICU bed. Fecal soilage/staining was used to verify that swabs were adequate. With the patient in the left lateral decubitus position, flocked nylon swabs (Eswab, Copan Diagnostics, Murrieta, CA) were inserted at least 6 cm into the rectum and spun clockwise five times while moving them longitudinally.

### Cultures for multi-drug resistant organisms

The organisms of interest in this study were MRSA, VRE, and MDR or Ceph-R Gram-negative bacteria. These organisms were selected because they are readily culturable and cause up to 40% of all culture-proven infections in the ICU [30]. Aliquoted rectal swab specimens were plated onto chromogenic agars for MRSA (Spectra MRSA agar, Remel, San Diego, CA)and VRE (Spectra VRE agar, Remel, San Diego, CA)), as well MacConkey agar for Gram-negative bacteria. Gram-negative colonies were selected from MacConkey based on predominant colony morphology, with identification and antimicrobial susceptibility testing performed using the Vitek 2 system with AST-N010/020 cards with confirmatory testing as needed. Current Clinical and Laboratory Standards Institute (CLSI) breakpoints were used for categorical interpretations of susceptibility. For this study, Gram-negative bacteria were classified as MDR if they were non-susceptible to three or more antimicrobial categories [31, 32]. Gram-negative bacteria were classified as Ceph-R if they showed non-susceptibility to 3rd generation cephalosporins (ceftriaxone or ceftazidime) [33]. All organisms of interest were classified as present or absent at ICU admission and again after 72 h.

### Early acquisition of gut pathogen colonization

Early acquisition of gut pathogen colonization was defined categorically, in a pathogen-specific manner for each individual patient. For the organisms of interest—MRSA, VRE, and the Gram-negative bacteria—patients were defined as having acquired gut pathogen colonization if the organism was absent at the time of ICU admission but present after 72 h.

### Clinical risk factors for early acquisition of gut pathogen colonization

We were interested in testing clinical variables as potential risk factors for early acquisition of gut pathogen colonization. The APACHE IV score was used to assess acute severity of illness, and was calculated at the time of ICU admission and again after 72 h. APACHE-IV is calculated by incorporating a comprehensive set of variables including age, sex, admission date to the ICU, vital signs, laboratory findings, and various clinical parameters to assess the severity of illness in critically ill patients. APACHE-IV scores were grouped into three categories: low (≤ 42), middling (43–73), and high (> 73). Besides assessing the total APACHE IV score, the component variables of the APACHE IV were also measured. We gathered the following co-variables in addition to the APACHE component variables: age (organized into tertiles), sex (biological sex at birth), ICU type (cardiac, medical, neurological, or surgical), ICU admission diagnosis, and pre-ICU hospital days. ICU admission diagnosis was determined by the primary reason for ICU admission, categorized by organ system. For all variables included in the APACHE score, we adopted the respective cutoffs specified by the APACHE criteria.

### Clinical outcomes: death or culture-proven infection

To examine the downstream impact of gut pathogen colonization, we examined a composite outcome of death and culture-proven infection within 30 days from ICU admission, an outcome that we and others have utilized previously [2]. The rationale for this composite outcome is that death and culture-proven infection function as competing risks because a patient might die from infection before the infection can be diagnosed. Operationalization of this outcome followed previously described methods.

### Statistical approach

Continuous measures were divided into tertiles. An exact binomial calculation was used to estimate the 95% confidence intervals for proportions. Chi-squared tests were used to compare co-variables based on early acquisition of gut pathogen colonization. A logistic regression model was constructed for the outcome of early acquisition of gut pathogen colonization. APACHE IV score was included in this model *a priori*, with additional predictor variables added stepwise and retained in the model if they had an independent association (*p* < 0.10) with the outcome. An additional model was examined which interrogated the component measures within APACHE IV as predictors. To examine the relationship between early acquisition of gut pathogen colonization and the composite outcome of death and culture-proven infection, we constructed a Cox proportional hazards model. This model had three exposure groups: patients who were not colonized at either ICU admission or 72 h later, patients who were colonized at ICU admission (with or without colonization at 72 h), and patients who were not colonized at admission but acquired one or more pathogens after 72 h. Last, a sample size calculation was performed to guide future ICU trials; this calculation was performed as a two-sided test of two proportions with alpha 0.05.

## Results

### Patient baseline characteristics

177 patients donated a rectal swab at ICU admission including 15 (8.5%) who died before 72 h had elapsed and 31 (17.5%) who remained alive but declined to provide a 72 h sample. This left 131 (74%) patients who survived 72 h and donated a second rectal swab for the analyses. Among the 131 patients, most were over 50 years old, with approximately equal numbers of females and males (Table 1). Patients were admitted to a variety of ICU types (medical, surgical, etc.) and were predominantly admitted directly to the ICU from the emergency room (74.0%). Acute severity of illness was high, with a median APACHE IV score of 52 (IQR 38 to 89).

**Table 1:** Baseline characteristics at the time of ICU admission, stratified by early gut colonization

Characteristics at the Time of ICU Admission		No Early Gut Colonization *N* = 104	Early Gut Colonization *N* = 27	*P*-value
Age (tertiles)	≤ 56 years	30 (75.0%)	10 (25.0%)	0.177
	57–67 years	39 (88.6%)	5 (11.4%)	
	≥ 68 years	35 (74.5%)	12 (25.5%)	
Sex	female	59 (79.7%)	15 (20.3%)	0.913
	male	45 (78.9%)	12 (21.1%)	
ICU Type	Cardiac	23 (82.1%)	5 (17.9%)	0.884
	Medical	31 (77.5%)	9 (22.5%)	
	Neurological	11 (73.3%)	4 (26.7%)	
	Surgical	39 (81.3%)	9 (18.8%)	
Admission Diagnosis, by System	Cardiovascular	35 (85.4%)	6 (14.6%)	0.735
	Digestive	28 (73.7%)	10 (26.4)	
	Respiratory	18 (75.0%)	6 (25.0%)	
	Genitourinary	7 (100%)	0 (0.0%)	
	Neurologic	6 (75.0%)	2 (25.0%)	
	Neurosurgery	5 (71.4%)	2 (28.6%)	
	Metabolic	1 (100%)	0 (0.0%)	
	Other	4 (80.0%)	1 (20.0%)	
Pre-ICU^1^ Days in Hospital	0 days	78 (80.4%)	19 (19.6%)	0.719
	1–2 days	13 (72.2%)	5 (27.8%)	
	> 2 days	13 (81.3%)	3 (18.8%)	
Receiving Dialysis	Yes	2 (66.7%)	1 (33.3%)	0.582
	No	102 (79.7%)	26 (20.3%)	
Receiving Ventilation	Yes	38 (84.4%)	7 (15.6%)	0.301
	No	66 (76.7%)	20 (23.3%)	
Vital Signs	Temperature > 38 °C	1 (50.0%)	1 (50.0%)	0.300
	Heart Rate ≥ 120/min	8 (80.0%)	2 (20.0%)	0.960
	Resp. Rate > 20/min	32 (76.2%)	10 (23.8%)	0.534
	MAP ≤ 65	9 (81.8%)	2 (18.2%)	0.835
Lab values	WBC (10^9^/L) > 10	63 (81.8%)	14 (18.2%)	0.412
	Hct (%) ≤ 40	81(77.9%)	23 (22.1%)	0.403
	Albumin (g/dL) ≤ 3.4	43 (78.2%)	12 (21.8%)	0.771
	Creatinine (mg/dL) > 1.2	37 (82.2%)	8 (17.8%)	0.562
Glasgow coma scale	< 5	27 (79.4%)	7 (20.6%)	0.973
	5–10	10 (76.9%)	3 (23.1%)	
	> 10	67 (79.8%)	17 (20.2%)	
APACHE IV score (tertiles)	Low (≤ 42 points)	35 (79.5%)	9 (20.5%)	0.842
	Middling (43–73 points)	36 (81.8%)	8 (18.2%)	
	High (> 73 points)	33 (76.7%)	10 (23.3%)	

ICU: Intensive Care Unit; APACHE IV: Acute Physiology and Chronic Health Evaluation IV

### Gut pathogen colonization

**Fig. 1:**
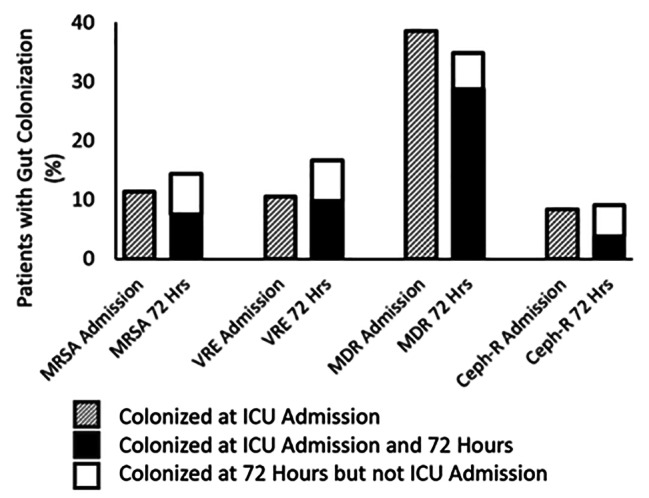
Proportion of patients with gut pathogen colonization at the time of ICU admission and 72 h later Stacked bar graph showing the proportions of patients colonized at ICU admission and again at 72 h. The bar graph is organized by organism and to highlight the patients who acquired colonization (white bars), meaning that they were colonized at 72 h but not at ICU admission. MRSA: methicillin-resistant *Staphylococcus aureus*; VRE: Vancomycin-Resistant Enterococcus; MDR: Multidrug Resistance; Ceph-R: Third Generation Cephalosporin-resistant

Rates of gut colonization at ICU admission were 11.4%, 10.6%, 38.6%, and 8.3% for MRSA, VRE, MDR Gram-negatives, and Ceph-R Gram-negatives respectively (Fig. 1). 48.1% of patients were colonized with one or more of these organisms at ICU admission. After 72 h in the ICU, rates of gut colonization were 14.4%, 16.7%, 34.8%, and 9.1% for the same organisms and 51.1% of patients were colonized with one or more organisms. Among 116 patients who were negative for MRSA at ICU admission, rates of early gut MRSA acquisition were 9/116 (7.8%, 95% CI 3.6 to 14.2%). Similarly, rates for early acquisition of VRE were 9/117 (7.7%, 95% CI 3.6 to 14.1%); rates for early acquisition of MDR Gram-negatives were 9/80 (11.3%, 95% CI 4.4 to 18.8%); rates for early acquisition of Ceph-R Gram-negatives were 5/120 (4.2%, 95% CI 1.4 to 9.5%). 18/131 (17.6%) of patients who tested positive for a given organism at the time of ICU admission had subsequently tested negative for the same organism at 72 h. For individual organisms, the rates were 5/131 (3.8%) for MRSA, 1/131 (0.8%) for VRE, 13/131 (9.9%) for MDR Gram-negatives, and 4/131 (3.1%) for Ceph-R organisms.

### Characteristics of colonizing Gram-negatives

**Fig. 2:**
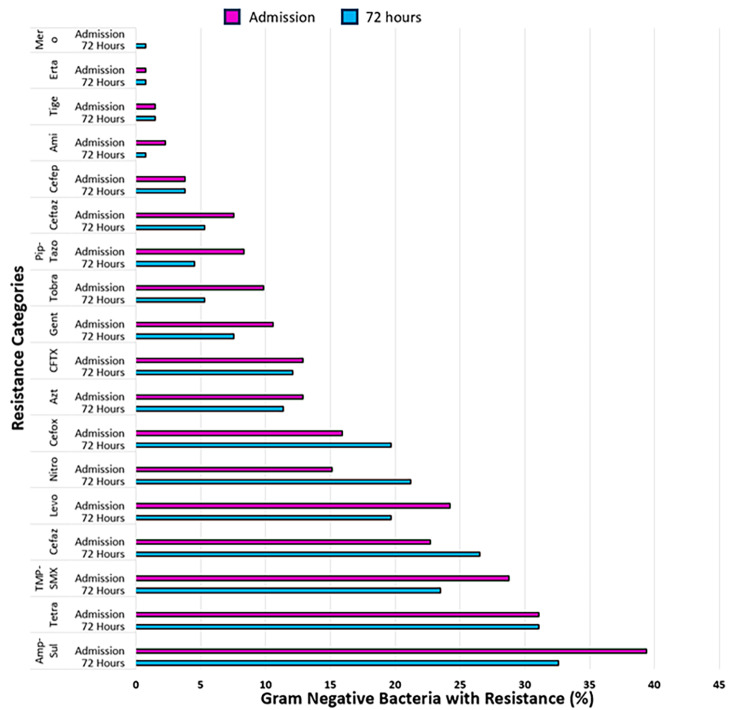
Antibiotic resistance pattern among Gram-negative bacteria cultured at the time of ICU admission and 72 h later Mero: Meropenem; Erta: Ertapenem; Tige: Tigecycline; Ami: Amikacin; Cefep: Cefepime; Ceftaz: Ceftazidime; Pip-Tazo: Piperacillin/Tazobactam; Tobra: Tobramycin; Gent: Gentamicin; CFTX: Ceftriaxone; Azt: Aztreonam; Cefox: Cefoxitin; Nitro: Nitrofurantoin; Levo: Levofloxacin; Cefaz: Cefazolin; TMP-SMX: Trimethoprim/Sulfamethoxazole; Tetra: Tetracycline; Amp-Sul: Ampicillin/Sulbactam

The most common Gram-negative bacteria cultured from rectal swabs were *E. coli*, followed by *K. pneumoniae* and *Enterobacter spp.* (Table 2). There were also 3 cases of non-MDR *Pseudomonas spp*. The comparison of the antimicrobial resistance patterns within Gram-negative bacteria showed minimal differences comparing the overall resistance pattern at ICU admission versus 72 h later (Fig. 2). The largest increase in resistance was observed with nitrofurantoin, followed by cefoxitin and cefazolin. However, the proportion of Gram-negatives showing non-susceptibility was similar for most antibiotics over this short timeframe.

**Table 2:** Gram-negative bacteria cultured from rectal swabs at ICU admission and 72 h later

Gram-Negative Organisms	Admission N (%)	72 h N (%)
*E. coli*	94 (71.2%)	83 (62.9%)
*K. Pneumoniae*	21 (21.2%)	24 (18.2%)
*Enterobacter spp.*	3 (2.3%)	11 (8.3%)
*Citrobacter spp.*	3 (2.2%)	6 (4.5%)
*Proteus spp.*	1 (0.8%)	4 (3.0%)
*S. Fonticola*	1 (0.8%)	2 (1.5%)
*Shigella spp.*	2 (1.5%)	1 (0.8%)
*H. Alvei*	1 (0.8%)	1 (0.8%)
*Burkholderia spp.*	0 (0.0%)	1 (0.8%)
*Raoutella spp.*	1 (0.8%)	0 (0.0%)
*Morganella spp.*	1 (0.8%)	0 (0.0%)

### Clinical risk factors for acquisition of early gut pathogen colonization

Baseline patient characteristics including acute severity of illness (APACHE IV) at ICU admission were compared between those who did versus those who did not acquire early gut pathogen colonization (Table 1). None of these clinical risk factors was associated with acquisition of early gut pathogen colonization. Neither APACHE IV score nor any other co-variables from the time of ICU admission predicted early gut pathogen colonization (odds ratio (OR) 0.86, 95% CI 0.30–2.49 for a middling vs. low APACHE IV score and OR 1.18, 95% CI 0.43–3.26 for a high vs. low APACHE IV score). In a multivariable model, there were no risk factors identified which associated with the outcome of early acquisition of pathogen colonization (Supplemental Table 1).

### Death and culture-proven infection

There were 11 deaths (8.4%) and 21 (16.0%) culture-proven infections within 30 days of ICU admission; 29 (22.1%) of patients had the combined outcome of death or infection within 30 days. The most common infection sites were urine (*N* = 10), respiratory (*N* = 7), and bloodstream (*N* = 7) (Table 3). The most common infection-causing organisms were *Klebsiella spp.* (*N* = 5) followed by *E. coli*, *Pseudomonas spp.*, and *Enterobacter spp.* (*N* = 4 each). Out of the 5 patients with Klebsiella infections, 1 was previously colonized with *Klebsiella spp.* (1/5, 20%), 4/4 (100%) were previously colonized with *E. coli*, and 0/4 (0%) were previously colonized with *Enterobacter spp*.

**Table 3:** Culture-proven infections within 30 days of ICU admission, organized by infection source and organism type

Infection Source	Total	Organism										
		*E. coli*	*Klebs-iella spp.*	MRSA	*Entero-bacter spp.*	*Pseudomonas spp.*	*E. faecalis*	VRE	*Citro-bacter spp.*	*Candida*	*A. xylox-idans*	MSSA
**Respiratory**	**7**	0	0	2	2	1	1	0	0	0	1	0
**Biliary Fluid**	**1**	0	1	0	0	0	0	0	0	0	0	0
**Urine**	**10**	2	2	0	1	1	2	1	1	0	0	0
**Blood**	**7**	0	2	0	1	0	0	1	0	1	0	1
**Wound**	**4**	1	0	0	0	1	1	1	0	0	0	0
**Abdominal Fluid**	**1**	1	0	0	0	0	0	0	0	0	0	0
**Totals**	**29**	**4**	**5**	**2**	**4**	**3**	**4**	**3**	**1**	**1**	**1**	**1**

### Acquisition of early gut pathogen colonization and risk for death or infection

Patient characteristics after 72 h in the ICU were examined to explore their association with subsequent occurrences of death or infection (Table 4). Longer pre-ICU hospital stays (> 2 days), receipt of dialysis, and receipt of mechanical ventilation, was associated with increased risk for the composite outcome. Elevated heart rate, respiratory rate, and creatinine levels were also associated with higher rates of death or infection, as was lower Glasgow Coma Scale scores (< 5 points) and higher APACHE IV score (Supplemental Table 2). Neither receipt of antibiotics nor receipt of PPIs was associated with the composite outcome. An additional analysis examining all-cause death showed an association between the APACHE IV score and all-cause death (OR 11.4, 95% CI 1.37–94.3); several additional APACHE IV score component variables were also associated with all cause death including creatinine, ventilation status, and Glasgow coma scale. Acquisition of early pathogen colonization was not associated with all cause death (OR 0.84, 95% CI 0.17–4.16).

**Table 4:** Characteristics after 72 h in the ICU, stratified by subsequent death or culture-proven infection

Characteristics after 72 h		No Death or Infection *N* = 102	Death or Infection *N* = 29	*P*-value
Age (tertiles)	≤ 56 years	29 (72.5%)	11 (27.5%)	0.612
	57–67 years	35 (79.5%)	9 (20.5%)	
	≥ 68 years	38 (80.9%)	9 (19.1%)	
Sex	female	60 (81.1%)	14 (18.9%)	0.312
	male	42 (73.7%)	15 (26.3%)	
ICU Type	Cardiac	24 (85.7%)	4 (14.3%)	0.402
	Medical	28 (70.0%)	12 (30.0%)	
	Neurological	11 (73.3%)	4 (26.7%)	
	Surgical	39 (81.3%)	9 (18.8%)	
Admission Diagnosis, by System	Cardiovascular	38 (92.7%)	3 (7.3%)	0.108
	Digestive	28 (73.7%)	10 (26.3%)	
	Respiratory	17 (70.8%)	7 (29.2%)	
	Genitourinary	5 (71.4%)	2 (28.6%)	
	Neurologic	6 (75.0%)	2 (25.0%)	
	Neurosurgery	4 (57.1%)	3 (42.9%)	
	Metabolic	0 (0.0%)	1 (100.0%)	
	Other	4 (80.0%)	1 (20.0%)	
Pre-ICU Days in Hospital	0 days	82 (84.5%)	15 (15.5%)	0.007
	1–2 days	10 (55.6%)	8 (44.4%)	
	> 2 days	10 (62.5%)	6 (37.5%)	
Receiving Dialysis	Yes	4 (44.4%)	5 (55.6%)	0.012
	No	98 (80.3%)	24 (19.7%)	
Receiving Ventilation	Yes	11 (50.0%)	11 (50.0%)	< 0.001
	No	91 (83.5%)	18 (16.5%)	
Vital Signs	Temperature > 38 °C	0 (0.0%)	1 (100.0%)	0.060
	Heart Rate ≥ 120/min	7 (70.0%)	3 (30.0%)	0.533
	Resp. Rate > 20/min	30 (69.8%)	13 (30.2)	0.119
	MAP ≤ 65	6 (60.0%)	4 (40.0%)	0.157
Lab values	WBC (10^9^/L) > 10	49 (73.1%)	18 (26.9%)	0.182
	Hct (%) ≤ 40	90 (75.6%)	29 (24.4%)	0.053
	Albumin (g/dL) ≤ 3.4	59 (73.8%)	21 (26.3%)	0.156
	Creatinine (mg/dL) > 1.2	33 (67.3%)	16 (32.7%)	0.025
Glasgow coma scale	< 5	2 (25.0%)	6 (75.0%)	< 0.001
	5–10	7 (58.3%)	5 (41.7%)	
	> 10	93 (83.3%)	18 (16.2%)	
APACHE IV score	≤ 42 points	59 (90.8%)	6 (9.2%)	< 0.001
	43–73	34 (73.9%)	12 (26.1%)	
	> 73	9 (45.0%)	11 (55.0%)	

ICU: Intensive Care Unit; APACHE IV: Acute Physiology and Chronic Health Evaluation IV

### Cox proportional hazards model for death or infection

**Fig. 3:**
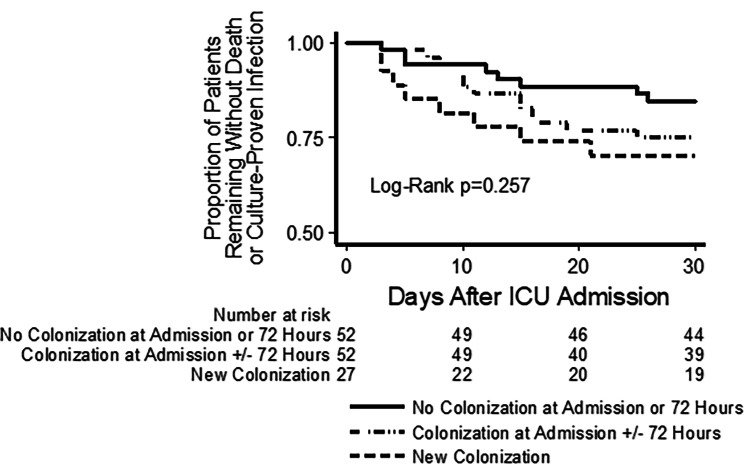
Kaplan-Meier plot showing the relationship between new gut pathogen colonization (within 72 h of ICU admission) and subsequent death or culture-proven infection within 30 days after ICU admission

Finally, we examined the relationship between new gut pathogen colonization and death or culture-proven infection using time-to-event methods (Fig. 3). Patients were divided into three groups: not colonized, colonized at admission (with or without colonization after 72 h), and new colonization. The highest rates of death or infection were among those who were newly colonized, followed by those with baseline colonization, and those without colonization had the lowest rates (Log-Rank not statistically significant, *p* = 0.257). Next, we constructed a Cox proportional hazards model for the outcome of death or culture-proven infection within 30 days of ICU admission. In this model, we compared three categories of patients: those who were never gut pathogen colonized, those who were colonized at ICU admission (regardless of colonization status after 72 h), and those who had early acquisition of gut pathogen colonization. Compared versus a reference group who was never colonized, the crude hazard ratio for death or infection was 1.69 (95% CI 0.70–4.09) for those who were colonized at ICU admission and was 2.19 (95% CI 0.82–5.84) for those who had early gut acquisition of colonization. APACHE IV score was a strong independent predictor for death or infection, but adjusting for APACHE score did not substantially alter the relationship between early gut colonization and death or infection.

## Discussion

This study assessed early acquisition of gut pathogen colonization by performing protocolized deep rectal swabs on patients at the time of ICU admission and exactly 72 h later. Focusing on acquisition of VRE, MRSA, or MDR/Ceph-R Gram-negative bacteria, we found that rates of early acquisition varied from 4.2 to 11.3% across the organisms of interest. Among patients who were not previously gut pathogen colonized, one in every five patients became colonized during this period of short-term follow-up. One caveat to this finding is that there was “noise” in our data, with some patients who were classified as colonized at ICU admission then testing negative three days later. This intermittent intestinal carriage has been observed in previous studies and may represent false negatives or true changes in carriage due to administration of antibiotics or natural clearance [8, 34, 35]. False negative misclassification of baseline swabs could lead to an overestimation of rates of early gut pathogen colonization.

Interestingly, the observed rates of 30-day death or infection were worse among patients who had early acquisition of gut pathogen colonization compared to those who were already colonized at the time of ICU admission, although this result was not statistically significant. Overall, our findings highlight the need for early interventions aimed at preventing acquisition of gut pathogen colonization in the ICU, particularly for patients at high risk for sepsis. The early hours of an ICU admission are a critical window during which interventions can have the most substantial impact on patient outcomes (e.g., in treatment of sepsis where in-hospital mortality can be reduced by early interventions by up to 16%) [36]. Our study demonstrates that substantial, likely clinically relevant, changes are taking place in terms of gut colonization status during this same early ICU time period.

In terms of specific organisms, our study results are similar to previous studies, although variation in the ICU population, local pathogen prevalence, and the timeframe of testing make exact comparisons challenging [3]. A prior meta-analysis estimated the acquisition rate of VRE to be 10.2% within the United States [18], which is similar to our finding of a VRE acquisition rate of 10.6% within the first 72 h. However, acquisition rates of VRE can vary greatly between studies based on frequency and type of screening [17, 37]. Our study had an MRSA acquisition rate of 7.8% during the initial 72 h of ICU admission and Thompson et al. similarly found an MRSA acquisition rate of 7.5%; however, this study and many others re-tested for colonization after one week or more instead of 72 h [38–40]. For Gram-negative bacteria, our study had a Ceph-R acquisition rate of 4.2% within the first 72 h of ICU admission which is similar to the 3% acquisition rate in the Americas estimated by a previous meta-analysis [3]. However, these studies screened weekly after admission [41, 42], so it is again unclear what proportion was acquired within 72 h. Given the similar rates of acquisition in our study after 72 h and other studies that screen after a greater time interval, it is likely that much pathogen acquisition takes place during the first few days after ICU admission.

Our results also can be used as the basis for sample size calculations for future trials seeking to intervene to decolonize patients or prevent colonization and emphasize that the appropriate design for future trials will depend on the hypothesized mechanism of the intervention. Pre-selection of patients based on rapid testing for colonization at the time of ICU admission is likely to enhance the efficiency of future trials design. Clinical variables, on the other hand, are less likely to be useful in pre-selecting patients for future trials; none of them—including APACHE IV score—were associated with acquisition of gut pathogens in this study. No clinical variables—including APACHE IV and its components—were associated with early gut colonization whereas APACHE score and a number of other clinical predictors were identified which associated with death or infection. Although no clinical variables were associated with early acquisition of gut pathogen colonization, we did not have access to data related to pre-ICU antibiotics exposure or to prior residence in a nursing home, two variables which might associate with colonization. Our single-center data is insufficient to conclusively determine whether clinical risk factors might be useful in pre-selecting colonized patients for trials.

This study has some strengths. The emphasis on early acquisition of gut pathogen colonization (within 72 h) is unusual. This approach provides unique insights into the rapid dynamics of gut colonization in critically ill patients, a timeframe that has been less explored in previous research. By narrowing the window of observation, the study highlights the urgency of addressing colonization during the initial stages of an ICU stay, which informs the timing of future interventions. Sample acquisition was strictly protocolized, was done in real time, and used low cost, readily accessible culture-based methods (as opposed to sequencing) that could be easily replicated across institutions. The study also has limitations. We did not have the resources to extend sample collection beyond 72 h; we have ongoing studies which will address this by taking protocolized samples on ICU days 0, 3, 7, 14, and 30. Future studies may wish to gather samples even more densely, and for longer durations. Our findings are derived from a diverse range of ICU types, but in a single institution, and the gut colonization characteristics of the background population are likely to influence ICU colonization and impact study generalizability. The decision to exclude subjects who were swabbed at ICU admission but did not undergo a second swab introduces a potential for immortal time bias. Last, the sample size was relatively small, with a consequent effect on the confidence for our estimates of acquisition of gut pathogen colonization.

In sum, this prospective cohort study found that over one in five ICU patients acquired gut pathogen colonization within 72 h of ICU admission (MRSA, VRE, or MDR/Ceph-R GN bacteria). By describing the dynamics of early gut pathogen colonization in the ICU, these results may guide future trials seeking to test ICU interventions to reduce or prevent gut pathogen colonization.

### Electronic supplementary material

Below is the link to the electronic supplementary material.

Supplementary Material 1

Supplementary Material 2

## Data Availability

The datasets used and/or analyzed during the current study are available from the corresponding author on reasonable request.

## Abbreviations

ICU: Intensive Care Unit
MRSA: Methicillin-Resistant Staphylococcus Aureus
VRE: Vancomycin-Resistant Enterococcus
MDR: Multidrug Resistance
Ceph-R: Third Generation Cephalosporin-resistant
APACHE IV: Acute Physiology and Chronic Health Evaluation IV
CRE: Carbapenem-Resistant Enterobacteriaceae
GN: Gram-negative
CLSI: Clinical and Laboratory Standards Institute
