# Supplementary material for: Timing and clinical risk factors for early acquisition of gut pathogen colonization with multidrug resistant organisms in the intensive care unit
Source: Gut Pathog. 2024 Feb 21;16:10. doi: 10.1186/s13099-024-00605-z (PMC10880254; doi:10.1186/s13099-024-00605-z)
Supplement: Supplementary file 1 — Supplementary Material 1 [file 13099_2024_605_MOESM1_ESM.docx]

| **Supplemental Table 1**. Risk factors for early acquisition of gut pathogen colonization. | | | |
| --- | --- | --- | --- |
| **Characteristics at the Time of ICU Admission** | | **Crude Odds Ratio**  **(95% CI)** | **Adjusted Odds Ratio (95% CI)** |
| Age (tertiles) | ≤ 56 years | Reference | --- |
|  | 57-67 years | 0.54 (0.18 – 1.63) | --- |
|  | ≥ 68 years | 1.17 (0.44 –2.12) | --- |
| Gender | female | 1.05 (0.45 – 2.46) | --- |
| ICU Type | Cardiac | Reference | --- |
|  | Medical | 1.34 (0.39 – 4.52) | --- |
|  | Neurological | 1.67 (0.37 – 7.48) | --- |
|  | Surgical | 1.06 (0.32 – 3.55) | --- |
| Admission Diagnosis, by System | Cardiovascular | Reference | --- |
|  | Digestive | 2.08 (0.67 – 6.43) | --- |
|  | Respiratory | 1.75 (0.50 – 6.16) | --- |
|  | Genitourinary | --- | --- |
|  | Neurologic | 1.94 (0.32 – 12.00) | --- |
|  | Neurosurgery | 2.33 (0.37 –14.90) | --- |
|  | Metabolic | --- | --- |
|  | Other | 2.92 (0.23 – 37.43) | --- |
| Pre-ICU Days in Hospital | 0 days | Reference | --- |
|  | 1-2 days | 1.58 (0.50 – 4.97) | --- |
|  | > 2 days | 0.95 (0.25 – 3.66) | --- |
| Receiving Dialysis | | 0.46 (0.06 – 3.86) | --- |
| Receiving Ventilation | | 0.56 (0.15 – 2.05) | --- |
| Vital Signs | Temperature >38°C | 4.08 (0.55-30.39) | --- |
|  | Heart Rate ≥120/min | 0.96 (0.19-4.81) | --- |
|  | Resp. Rate > 20/min | 1.55 (0.65-3.70) | --- |
|  | MAP ≤ 65 | 1.73 (0.42-7.20) | --- |
| Lab values | WBC (10^9^/L) > 10 | 1.51 (0.64-3.57) | --- |
|  | Hct (%) ≤ 40 | 1.33 (0.27-6.46) | --- |
|  | Albumin (g/dL) ≤ 3.4 | 0.76 (0.32-1.78) | --- |
|  | Creatinine (mg/dL) > 1.2 | 0.52 (0.20-1.33) | --- |
| Glasgow coma scale | < 5 | Reference | --- |
|  | 5-10 | 2.33 (0.20 – 27.57) | --- |
|  | > 10 | 1.83 (0.21 – 15. 63) | --- |
| APACHE IV score (tertiles) | Low (≤ 42 points) | Reference | Reference |
|  | Middling (43-73 points) | 0.86 (0.30-2.49) | 0.86 (0.30-2.49) |
|  | High (> 73 points) | 1.18 (0.43-3.26) | 1.18 (0.43-3.26) |

ICU: Intensive Care Unit; APACHE IV: Acute Physiology and Chronic Health Evaluation IV
