# Supplementary material for: Timing and clinical risk factors for early acquisition of gut pathogen colonization with multidrug resistant organisms in the intensive care unit
Source: Gut Pathog. 2024 Feb 21;16:10. doi: 10.1186/s13099-024-00605-z (PMC10880254; doi:10.1186/s13099-024-00605-z)
Supplement: Supplementary file 2 — Supplementary Material 2 [file 13099_2024_605_MOESM2_ESM.docx]

| **Supplemental Table 2**. Risk factors for death or infection. | | | | | | |
| --- | --- | --- | --- | --- | --- | --- |
| **Characteristics at 72 Hours** | | | **Crude Odds Ratio**  **(95% CI)** | | **Adjusted Odds Ratio (95% CI)** | |
| Early gut colonization | | |  | |  | |
| Age (tertiles) | ≤ 56 years | Reference | | --- | |  |
|  | 57-67 years | 0.59 (0.22-1.63) | | --- | |  |
|  | ≥ 68 years | 0.70 (0.26-1.90) | | --- | |  |
| Gender | female | 1.53 (0.67-3.5) | | --- | |  |
| ICU Type | Cardiac | Reference | | --- | |  |
|  | Medical | 2.57 (0.73-9.03) | | --- | |  |
|  | Neurological | 2.18 (0.46-10.37) | | --- | |  |
|  | Surgical | 1.38 (0.38-5.00) | | --- | |  |
| Admission Diagnosis, by System | Cardiovascular | Reference | | --- | |  |
|  | Digestive | 4.52 (1.14-17.97) | | --- | |  |
|  | Respiratory | 4.67 (1.08-20.10) | | --- | |  |
|  | Genitourinary | 5.07 (0.67-38.10) | | --- | |  |
|  | Neurologic | 4.22 (0.58-30.75) | | --- | |  |
|  | Neurosurgery | 9.5 (1.42-63.72) | | --- | |  |
|  | Metabolic | --- | | --- | |  |
|  | Other | 6.33 (0.44-91.71) | | --- | |  |
| Pre-ICU Days in Hospital | 0 days | Reference | | --- | |  |
|  | 1-2 days | 4.37 (1.48-12.88) | | --- | |  |
|  | > 2 days | 3.28 (1.04-10.38) | | --- | |  |
| Receiving Dialysis | | 5.10 (1.27-20.46) | | --- | |  |
| Receiving Ventilation | | 5.06 (1.90-13.43) | | --- | |  |
| Vital Signs | Temperature >38°C | 11.65 (1.16-116.69) | | --- | |  |
|  | Heart Rate ≥120/min | 1.57 (0.38-6.48) | | --- | |  |
|  | Resp. Rate > 20/min | 1.95 (0.84-4.55) | | --- | |  |
|  | MAP ≤ 65 | 2.56 (0.67-9.77) | | --- | |  |
| Lab values | WBC (10^9^/L) > 10 | 1.77 (0.76-4.12) | | --- | |  |
|  | Hct (%) ≤ 40 | --- | | --- | |  |
|  | Albumin (g/dL) ≤ 3.4 | 2.33 (0.95-5.75) | | --- | |  |
|  | Creatinine (mg/dL) > 1.2 | 2.57 (1.11-5.97) | | --- | |  |
| Glasgow coma scale | < 5 | Reference | | --- | |  |
|  | 5-10 | 0.24 (0.03-1.71) | | --- | |  |
|  | > 10 | 0.06 (0.01-0.35) | | --- | |  |
| APACHE IV score (tertiles) | Low (≤ 42 points) | Reference | | Reference | |  |
|  | Middling (43-73 points) | 2.04 (0.62-6.70) | | 2.04 (0.62-6.70) | |  |
|  | High (> 73 points) | 4.16 (1.35-12.84) | | 4.16 (1.35-12.84) | |  |
